# A Case of Otomyiasis in an Intoxicated Patient

**DOI:** 10.7759/cureus.97352

**Published:** 2025-11-20

**Authors:** Amanda T Tran, Daniel T Lee, Jetrina Maque

**Affiliations:** 1 Family Medicine, University of California Los Angeles David Geffen School of Medicine, Los Angeles, USA; 2 Internal Medicine, University of California Los Angeles, Los Angeles, USA

**Keywords:** aural myiasis, case report, maggots, otalgia, otomyiasis

## Abstract

Otomyiasis, or aural myiasis, is a parasitic disease caused by the infestation of the external and/or middle ear by maggots, the larval stage of flies. It is a rare condition that is most often seen in developing countries, particularly in tropical and rural areas. In this report, we describe a rare case of otomyiasis in an urban setting in the United States in a patient with alcohol use disorder. This case report highlights the importance of vigilance in individuals with predisposing factors who present with ear symptoms.

## Introduction

Myiasis is a parasitic disease caused by the infestation of maggots, the larval stage of flies, in human tissue [1]. The disease begins when a fly lays eggs on an open wound, intact skin, or an external orifice [2]. Larvae hatching from the eggs can penetrate the tissue, thereby leading to different symptoms based on the affected organ. In particular, otomyiasis, or aural myiasis, occurs when larvae infest the external and/or middle ear. Otomyiasis is a rare condition, with only 45 cases reported globally over a 20-year period according to a comprehensive literature review from 2015 [3]. Another systematic review found that most cases were in developing countries, especially in tropical regions and rural areas [4]. Only four cases were reported in the United States from 1994 to 2014, and all four of these cases were associated with malignancy [5].

Here, we present a rare case of otomyiasis in an urban setting in the United States in a patient with alcohol use disorder. This case report highlights the need for vigilance in at-risk patients.

## Case presentation

A 48-year-old male patient with a past medical history of alcohol use disorder and financial insecurity was brought in by ambulance to the Emergency Department for a closed head injury after being found down in the street in the summertime. The patient reported that he had a ground-level fall while intoxicated. Further history was unable to be obtained from the patient due to his somnolence and altered mental status. His ethyl alcohol and phosphatidylethanol levels were consistent with severe acute intoxication and chronic heavy alcohol use, respectively (Table 1). His urine drug screen was negative for other recreational substances. A computerized tomography scan of his brain showed no abnormalities. The patient was admitted for monitoring of his neurologic status.

**Table 1:** Serum alcohol test results

Test	Result	Reference range
Ethyl alcohol	489 mg/dL	< 15 mg/dL
Phosphatidylethanol	> 2000 ng/mL	< 10 ng/mL

**Figure 1:**
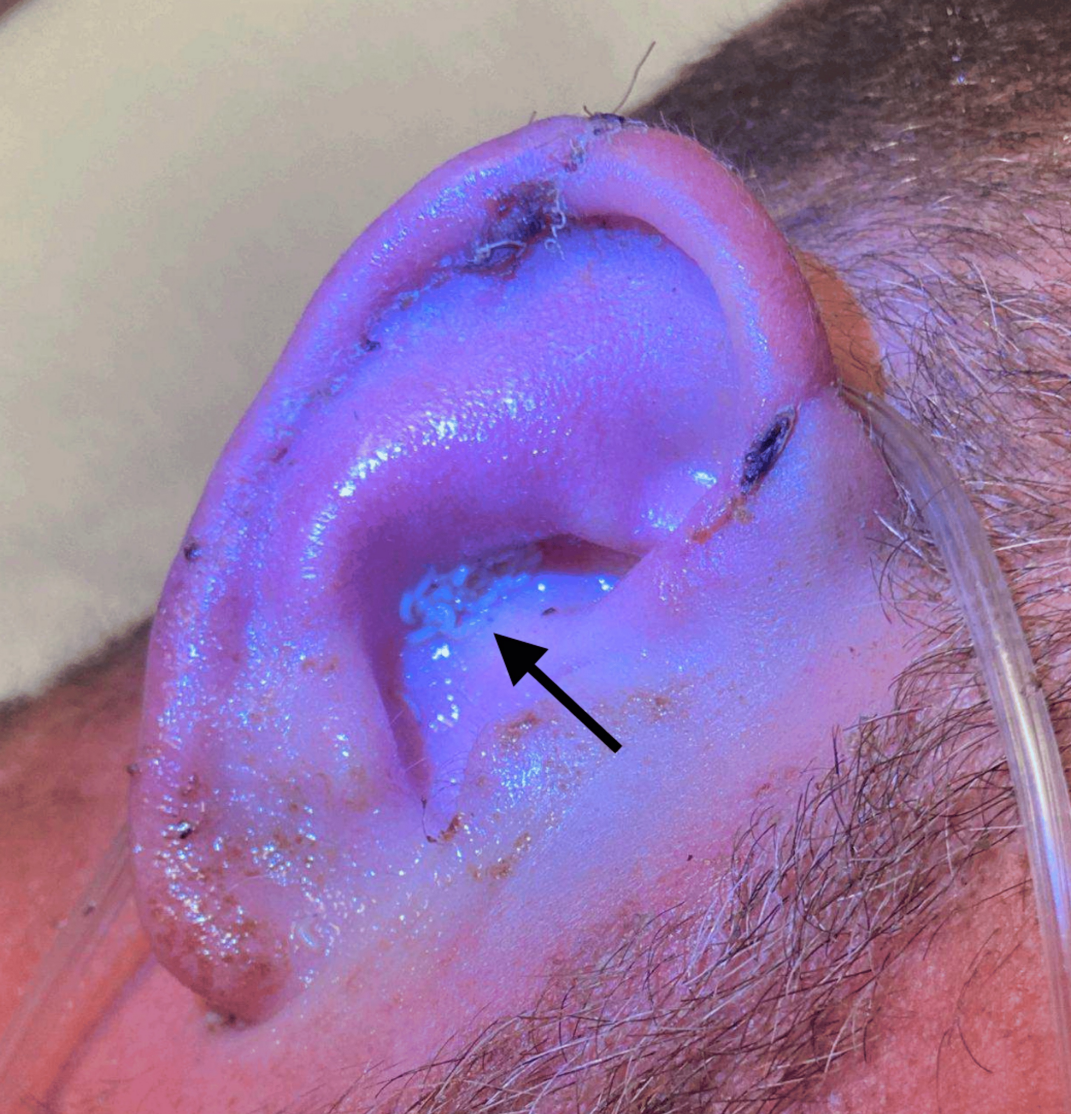
Mobile larvae (black arrow) in the patient’s right external auditory canal

The patient’s nurse reported that he would intermittently scream and hit his right ear. On gross visual inspection, the patient had mobile larvae occluding his right external auditory canal (Figure 1). Sterile mineral oil was instilled in the right external auditory canal for 10 minutes in an attempt to suffocate the larvae and induce their migration out of the ear canal. The ear was abundantly irrigated with saline solution 0.9%, yielding “several dozen” larvae per the nursing report. Otoscopic examination after the saline irrigation revealed numerous densely mobile larvae still obstructing the view of the tympanic membrane.

**Figure 2:**
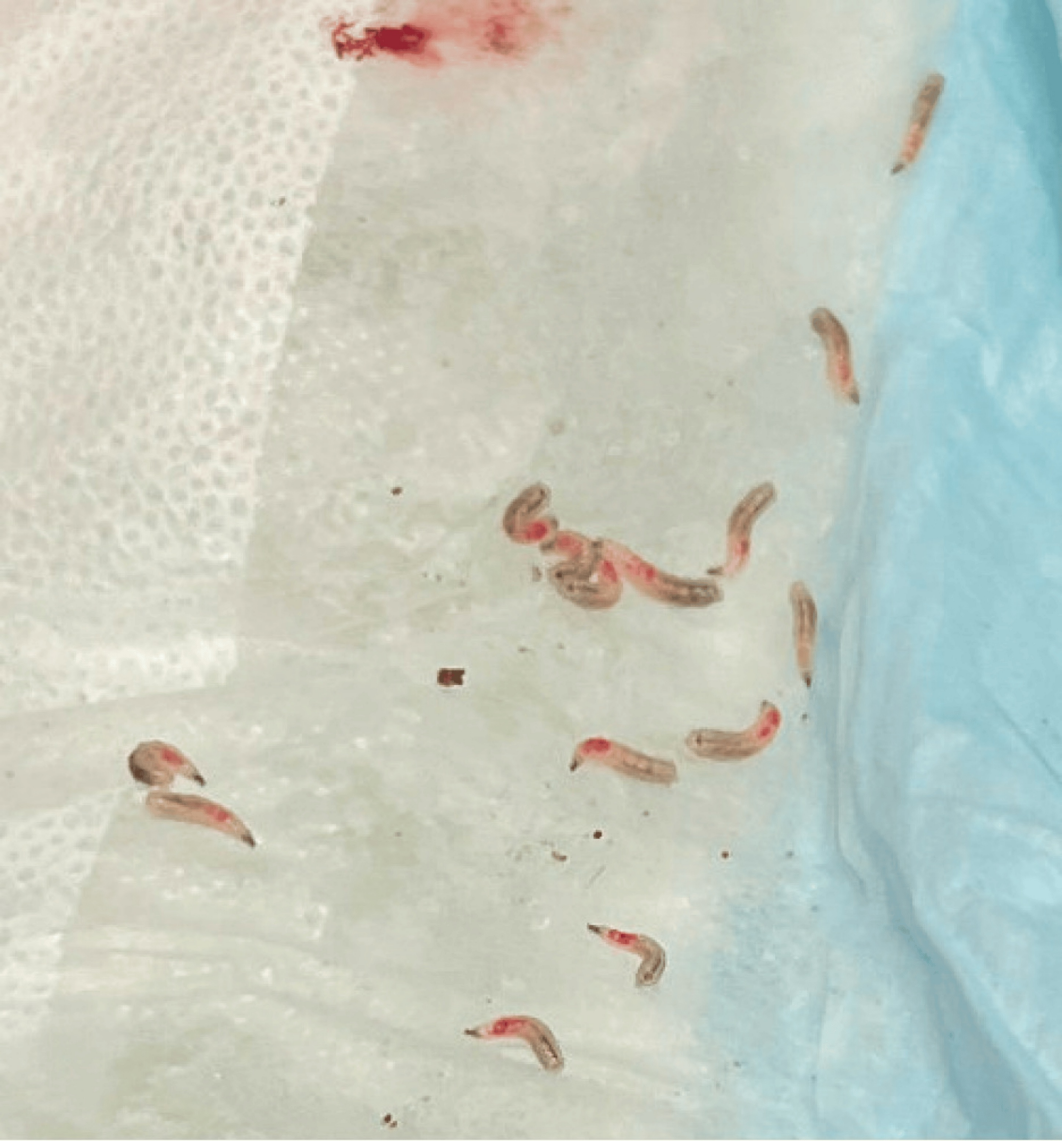
Multiple larvae extracted from the patient’s right ear

The following morning, the patient was alert and fully oriented. He reported right ear pain, bloody ear discharge, and a “crackling” sensation in the ear. He was unable to recall whether he had ear symptoms prior to this hospitalization. Consent was obtained, and the otolaryngology team was consulted for manual extraction of the larvae. Topical lidocaine was applied to the right external auditory canal to minimize local irritation. A total of 26 larvae were individually extracted with alligator forceps (Figure 2). The ear was circumferentially suctioned, and the tympanic membrane was visualized and intact, with no further larvae appreciated. The remaining clinical exam was consistent with acute otitis media and externa. There was no mastoid tenderness to palpation. An additional saline lavage was performed 12 hours post-extraction in case of residual egg hatching, with removal of one additional larva from the ear. The patient was treated with a seven-day course of oral amoxicillin-clavulanate and ciprofloxacin-dexamethasone otic drops. Subsequent otoscopic examinations confirmed no further infestation and improvement in the secondary ear infections. The patient was scheduled for an outpatient follow-up appointment one week after hospital discharge; however, he was, unfortunately, lost to follow-up. In addition, several attempts were made to contact the patient on his cell phone, but were also unsuccessful.

## Discussion

Although otomyiasis is rare in the United States, it is an important medical condition to consider in individuals with risk factors who present with ear symptoms. Some predisposing factors for otomyiasis include chronic otitis media, previous otologic surgery, immunosuppression, neurologic and psychosocial disorders, substance use, low socioeconomic status, poor personal hygiene, and immobility [6]. In this case, the infestation in this patient with financial insecurity was thought to be facilitated by the prolonged period of immobility due to the alcohol intoxication [7]. The female fly, which is generally attracted to normal and pathologic secretions from the ear, was likely able to lay numerous eggs in the patient’s external auditory canal due to the persistent decreased level of consciousness [8]. Additionally, the warm and humid environment during the summer likely fostered the maturation of the larvae [7].

The most common symptoms of otomyiasis are otalgia, otorrhea, pruritus, foreign body sensation, and aural fullness due to the mechanical effects of the larvae [9]. If an invasive species of larva infests an individual, they can secrete enzymes that enable them to liquefy and then feed on tissue. This feeding process can lead to complications such as tympanic membrane perforation, mastoid cavity invasion, and central nervous system penetration [2]. Thus, early diagnosis and treatment are pivotal in preventing these complications.

Otomyiasis is diagnosed by clinical examination. A computerized tomography scan is indicated if there is suspicion for complications other than tympanic membrane perforation [1]. While there is no standard treatment for otomyiasis, the goal of treatment is the removal of all larvae. In some cases, the larvae can be simply removed by copious irrigation of the ear with saline solution 0.9%, 70% ethanol, or povidone-iodine solution [10]. Suctioning can also be helpful in removing the larvae. If larvae still remain after irrigation and suctioning, the larvae should be manually extracted under micro-otoscopy using alligator forceps. It is important to avoid crushing the larvae in the ear while extracting them, as crushing can make complete removal difficult, and any dead organic material remaining in the ear can cause a foreign body reaction [11]. Therefore, topical ivermectin can be applied immediately before manual extraction to paralyze the larvae and aid in the extraction [1,12]. If topical ivermectin is not available, as in this case, an occlusive agent, such as mineral oil or petroleum jelly, can be applied instead. The production of localized hypoxia forces the larvae to migrate up to the surface to breathe, thereby facilitating the extraction [12]. Given that these solutions all achieve the same goal, the most effective topical adjunct is debatable [2]. A promising systemic adjunct is oral ivermectin, which has been shown to be effective in orbital and oral myiasis [5,8]. However, further research is needed, as there has been only one case report on the use of oral ivermectin for otomyiasis [13].

After removal of all the larvae, saline lavages 12 hours afterward is recommended in case of residual egg hatching. Topical and oral antibiotics are often prescribed prophylactically or to treat secondary infections [11]. If the tympanic membrane is perforated, an audiologic assessment should be performed to evaluate for hearing loss [10]. If mastoid cavity invasion or central nervous system penetration is suspected, surgical intervention is needed [9]. While there is no standard follow-up interval, one study recommends a follow-up otoscopic examination for all cases of otomyiasis within a week to confirm complete removal of larvae [14]. Follow-up care for patients with tympanic membrane perforation or invasive disease should also include a repeat audiologic assessment [10].

Due to the possible devastating complications of otomyiasis, public health measures should be implemented to reduce the risk and severity of this disease. For example, increasing awareness among vulnerable populations and health education among those caring for them may help prevent infestation. Furthermore, one prospective study advocates for myiasis to be a reportable disease in order to better understand its epidemiology. This study suggests that the larvae should be submitted to a clinical laboratory or a center of agriculture, entomology, parasitology, or vector control [14]. In doing so, the species of larva, its potential for invasiveness, and distribution can be identified. Mandatory reporting may also ensure that affected individuals receive appropriate medical treatment as well as social assistance if needed. Unfortunately, in this case, the larvae were not submitted to the laboratory, and it is not known whether the patient received appropriate social care after hospital discharge.

## Conclusions

In summary, otomyiasis is a rare parasitic disease in the United States caused by the infestation of the ear by fly larvae. While the initial clinical presentation typically involves localized ear symptoms, neurologic manifestations may emerge in the late stages of otomyiasis due to central nervous system penetration. Therefore, vigilance for otomyiasis is essential, particularly in those with predisposing factors such as low socioeconomic status, substance use, and immobility. Familiarity with treatment options is also critical. The management of otomyiasis includes aural irrigation, manual extraction, and empiric antibiotics. When the infestation extends beyond the tympanic membrane, prompt surgical intervention can be lifesaving. Thus, this case report underscores the need for increased awareness, education, and reporting of otomyiasis.
